# Psychosocial function moderates the link between sensory function and cognitive performance in individuals with mild cognitive impairment and Alzheimer's disease

**DOI:** 10.1002/alz70857_105917

**Published:** 2025-12-25

**Authors:** Sana Rehan, Fereshteh Mehrabi, Paul Mick, Natalie A. Phillips

**Affiliations:** ^1^ Concordia University, Montreal, QC, Canada; ^2^ University of Saskatchewan, Saskatoon, SK, Canada

## Abstract

**Background:**

Sensory and psychosocial factors are potentially modifiable risk factors for dementia. Social factors and indicators of mental health are associated with cognitive performance and risk for Alzheimer's disease (AD). Sensory impairment can lead to communication and mobility difficulties, reducing social engagement and possibly accelerating cognitive decline. We aimed to 1) characterize sensory and psychosocial function in groups with (or at risk for) AD and 2) test whether psychosocial factors and group membership moderate the sensory‐cognitive relationship.

**Method:**

We used data from the Comprehensive Assessment of Neurodegeneration and Dementia (COMPASS‐ND) study (Release 7) to assess sensory, psychosocial, and cognitive function in cognitively normal controls (*N* = 123, *M*_Age_=69.7, 76% female), individuals with mild cognitive impairment (MCI, *N* = 348, *M*_Age_=73.2, 42% female), or with mild AD (*N* = 151, *M*_Age_=75.1, 41% female). Sensory loss was measured using speech‐reception thresholds (hearing) and contrast sensitivity (vision). Psychosocial factors (depression, anxiety, quality of life, social support, social isolation) were assessed via self‐reported questionnaires. Cognitive function was evaluated using scores on neuropsychological tests (memory, executive function, verbal fluency, processing speed). Correlation analyses were done to assess sensory, psychosocial, and cognitive function for all groups. For moderated moderation analyses, we examined the three‐way interaction of psychosocial factors and diagnostic group on cognitive performance, controlling for age, sex, education, and income.

**Result:**

Hearing loss was more prevalent in those with MCI (42%) and AD (48%) compared to controls (27%), as was low contrast sensitivity (4% controls, 20% MCI, 37% AD). Compared to controls, individuals with MCI and AD reported worse psychosocial function (e.g., depression, anxiety, lower quality of life) and reduced social engagement. Moderation analyses indicated that poor psychosocial function (e.g., social isolation, depressive or anxiety symptoms) amplified the sensory‐cognitive association in individuals with MCI, compared to controls and those with AD.

**Conclusion:**

Psychosocial function moderates the relationship between sensory loss and cognitive function differently across diagnostic groups. Particularly, individuals with MCI show the strongest relationship between sensory and cognitive function at poor levels of psychosocial function. Our research explored sensory‐psychosocial‐cognitive interactions in groups with varying degrees of cognitive impairment, laying the groundwork for understanding how sensory loss and psychosocial deficits contribute to cognitive decline.

